# Diffusion tensor-based analysis of white matter in dogs with idiopathic epilepsy

**DOI:** 10.3389/fvets.2023.1325521

**Published:** 2023-12-18

**Authors:** Katrin M. Beckmann, Adriano Wang-Leandro, Frank Steffen, Henning Richter, Matthias Dennler, Rima Bektas, Ines Carrera, Sven Haller

**Affiliations:** ^1^Section of Neurology, Department of Small Animals, Vetsuisse Faculty Zurich, University of Zurich, Zurich, Switzerland; ^2^Graduate School for Cellular and Biomedical Sciences, University of Bern, Bern, Switzerland; ^3^Clinic for Diagnostic Imaging, Department of Diagnostics and Clinical Services, Vetsuisse-Faculty Zurich, University of Zurich, Zurich, Switzerland; ^4^Department of Small Animal Medicine and Surgery, University of Veterinary Medicine Hannover, Hannover, Germany; ^5^Section of Anaesthesiology, Department of Clinical Diagnostics and Services, Vetsuisse-Faculty Zurich, University of Zurich, Zurich, Switzerland; ^6^Vet Oracle Teleradiology, Norfolk, United Kingdom; ^7^Department of Surgical Sciences, Radiology, Uppsala University, Uppsala, Sweden; ^8^Faculty of Medicine, University of Geneva, Geneva, Switzerland

**Keywords:** TBSS, white matter integrity, seizures, Border Collie, Greater Swiss Mountain dog, MRI, DTI, canine

## Abstract

**Introduction:**

The understanding of epileptic seizure pathogenesis has evolved over time, and it is now generally accepted that not only are cortical and subcortical areas involved but also the connection of these regions in the white matter (WM). Recent human neuroimaging studies confirmed the involvement of the WM in several epilepsy syndromes. Neuroimaging studies investigating WM integrity with diffusion tensor imaging (DTI) in canine idiopathic epilepsy are lacking. This study aimed to test the hypothesis that WM diffusion changes can be found in dogs affected by idiopathic epilepsy.

**Method:**

Twenty-six dogs with idiopathic epilepsy (15 Border Collies and 11 Greater Swiss Mountain dogs) and 24 healthy controls (11 Beagle dogs, 5 Border Collies, and 8 Greater Swiss Mountain dogs) were prospectively enrolled. Most dogs with idiopathic epilepsy (17/26) were enrolled within 3 months after seizure onset. Diffusion tensor imaging of the brain with 32 diffusion directions (low b value = 0 s/mm^2^; maximal b value = 800 s/mm^2^) was performed in a 3 Tesla scanner. Tract-based spatial statistics (TBSS), a voxel-based approach, was used to investigate changes in fractional anisotropy (FA) and mean diffusivity (MD) in the idiopathic epilepsy group compared to the healthy control group. Additionally, FA and MD were investigated in the region of corpus callosum and cingulate white matter in both groups.

**Results:**

We observed subtle changes in WM DTI between the idiopathic epilepsy group and the healthy control group limited to cingulate WM, with a significantly lower FA in the idiopathic epilepsy group compared to the healthy control group in the region of interest (ROI) approach (*p* = 0.027). No significant changes were found between the idiopathic epilepsy group and the healthy control group in the TBSS analysis and in the corpus callosum in the ROI approach.

**Conclusion:**

This study supports the cingulate area as a target structure in canine epilepsy. The subtle changes only might be explained by the short duration of epilepsy, small sample sizes, and the higher variability in canine brain anatomy. Furthermore, all included dogs showed generalized tonic-clonic seizures, possibly affected by generalized epilepsy syndrome, which are also associated with less pronounced DTI changes in humans than focal epilepsy syndromes.

## 1 Introduction

Canine idiopathic epilepsy is diagnosed based on the age of the dog at the onset of the epileptic seizure, unremarkable inter-ictal physical and neurological examination, and exclusion of metabolic, toxic, and structural cerebral disorders by means of diagnostic investigations (1). Magnetic resonance imaging (MRI) of the brain is routinely used as an important diagnostic step for idiopathic epilepsy, and the diagnosis is based on a normal structural brain MRI (2). However, epilepsy is increasingly recognized as a disease of the brain network, involving both gray matter and white matter, which cannot be assessed from conventional MRI sequences, but only with advanced MRI techniques (3–5). Diffusion tensor imaging (DTI) offers a unique opportunity to investigate the white matter structures non-invasively *in vivo* (6).

In order to detect diffusion within tissues, magnetic field gradients are used to create an image that is sensitized to diffusion in a particular direction, and thus, diffusion can be measured by estimating a three-dimensional diffusion model or tensor (7). This tensor is characterized by three orthogonal vectors. The average diffusivity of all three vectors in DTI represents the mean diffusivity (MD) value (8). The MD can be used to measure the microstructural properties of the gray and the white matter and is dependent on the amount of extracellular water (7).

**Figure 1:**
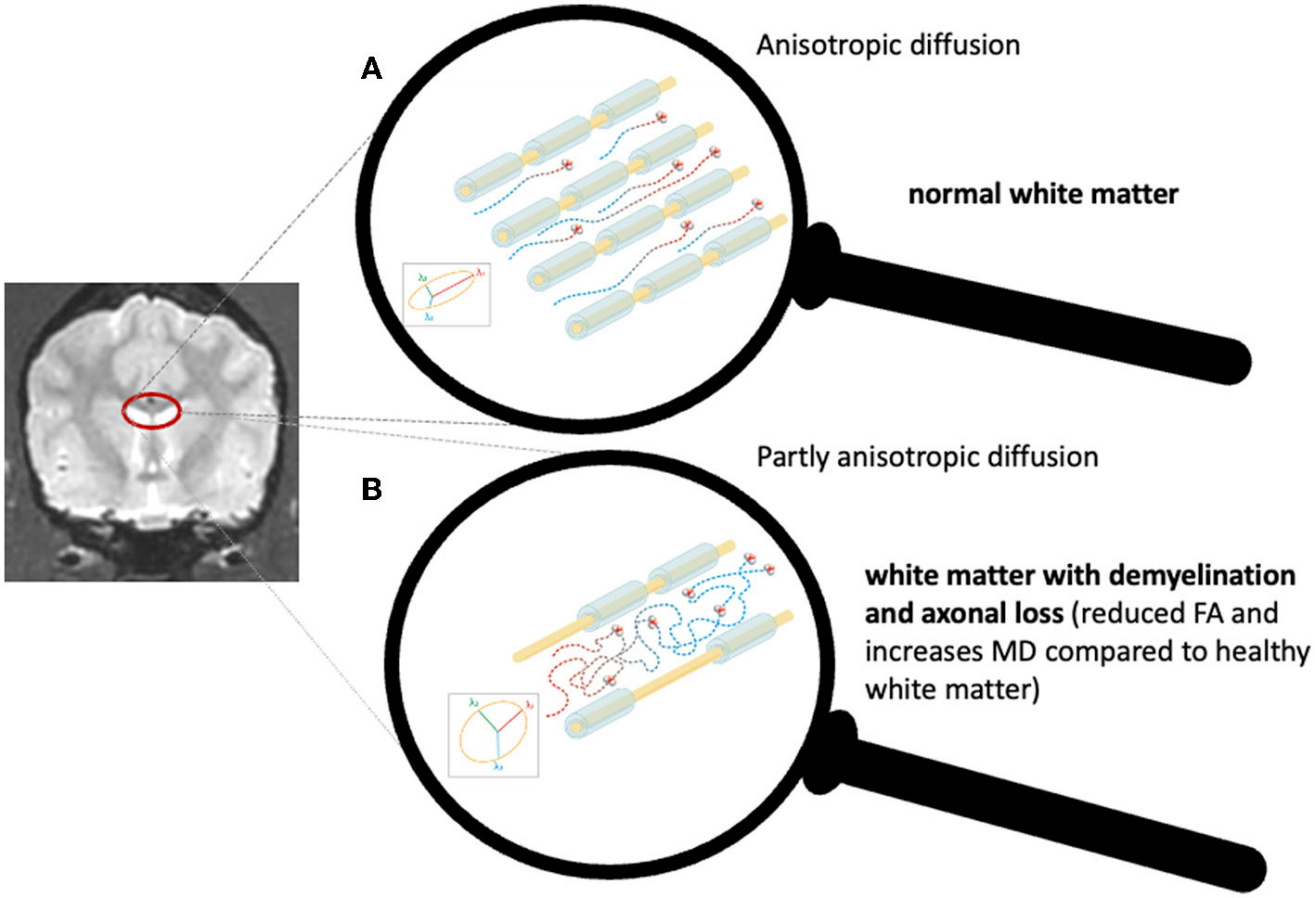
Schematic representation of normal water molecule diffusion along the direction of intact axonal bundles **(A)** and reduced anisotropy caused by axonal loss and demyelination **(B)**.

A preferential diffusion in a particular direction is called anisotropic diffusion. White matter is organized in tracts that consist of axonal bundles. The cellular membranes of these axons with some contributions from the myelination and the packing of the axons give a preferential direction of diffusion along the orientation of the axons leading to an anisotropic diffusion within the white matter (7). The most widely used metric for assessing anisotropy is fractional anisotropy (FA), and it is often considered a measure of white matter integrity (7). In cases of compromised white matter integrity, such as demyelination or axonal loss, reduced FA and increased MD values are expected (Figure 1).

In 2020, the ENIGMA Epilepsy study investigated DTI data of 1,249 human patients affected by a variety of epilepsy syndromes, including temporal lobe epilepsy, genetic generalized epilepsy, and non-lesional extratemporal epilepsy (5). Across all these epilepsy syndromes, the FA was lower in most fiber tracts. This effect was most prominent in the corpus callosum, cingulum, and external capsule. The reduction in FA was accompanied by a less robust increase in MD (5).

So far, a single study has been performed in veterinary medicine to investigate diffusion changes in dogs with idiopathic epilepsy. This study focused on apparent diffusion coefficient (ADC) metrics, a technique that describes the overall diffusion within a voxel but lacks a tensor imaging technique (9). Although DTI of the canine brain has been used to detect age-related changes (10), to detect differences between humans and dogs in Krabbe's disease (11), and to investigate the white matter in a compulsive behavioral disorder in dogs (12), the involvement of microstructural white matter changes in canine idiopathic epilepsy remains an unexplored field. Characterizing microstructural white matter changes, which are otherwise undetected in conventional MRI, could release the potential of establishing prognostic non-invasive biomarkers or objective quantitative monitoring parameters for the brain tissue in patients treated with novel treatment strategies such as epilepsy surgery or deep brain stimulation (13, 14).

The study aimed to investigate white matter diffusion changes in dogs affected by idiopathic epilepsy with generalized tonic-clonic seizures. We hypothesized that dogs with idiopathic epilepsy would have lower FA and higher MD in several white matter tracts compared to healthy controls and that this effect would be most pronounced in the corpus callosum and cingulate white matter.

## 2 Methods

### 2.1 Animals

Border Collies and Greater Swiss Mountain dogs diagnosed with idiopathic epilepsy according to the veterinary epilepsy task force criteria (15) and healthy controls of the same breeds were prospectively enrolled in this study during a period of 5 years (2017–2022). Additionally, 11 research Beagles were included in the healthy control group. These Beagle dogs have been part of a preliminary study investigating the feasibility of resting state network detection under general anesthesia (16). Functional MRI and magnetic resonance spectroscopy data from the same scan of most of the Border Collies and Greater Swiss Mountain dogs with idiopathic epilepsy and healthy controls have also been analyzed in previous studies (17–19). This study was approved by the Swiss Federal Veterinary Office Zurich (animal license numbers ZH272/16 and ZH046/20). The authors complied with the Animal Research: Reporting of *In Vivo* (ARRIVE Experiments) guidelines.

A physical and neurological examination was conducted by a board-certified veterinary neurologist in all dogs. Minimum database information was collected as recommended by the international veterinary epilepsy task force (IVETF) for investigations of idiopathic epilepsy, including a seizure history and family seizure history for idiopathic epilepsy, complete blood cell count, and serum biochemistry panel, electrolytes as well as fasted ammonia, bile acids, and urinalysis (1). Information on seizure semiology was collected from all owners in a personal interview based on the questionnaire recommended by the IVETF (1, 15).

Border Collies and Greater Swiss Mountain dogs with idiopathic epilepsy were included only, if they fulfilled the IVETF Tier II criteria and suffered from generalized tonic-clonic seizures (1). Healthy controls were included if they had no history of seizures.

Exclusion criteria were age younger than 1 year [to exclude the influence of incomplete maturation (20)] and older than 10 years [to exclude changes from aging (10)], DTI of insufficient quality, abnormal clinical or neurological examination, abnormal cerebrospinal fluid analyses, and identification of an underlying cause for the epilepsy or structural brain lesion on MRI.

Dogs were divided into two groups: the healthy control group and the idiopathic epilepsy group.

For the region of interest (ROI) analysis, the healthy control group was further subdivided into a healthy control group with a first-degree relative with idiopathic epilepsy and a healthy control group with no family history of idiopathic epilepsy.

### 2.2 Imaging acquisition

All dogs underwent an MRI scan of the brain with a 3 Tesla MRI (Philips Ingenia scanner, Philips AG, Zurich, Switzerland) and a 16-channel receive–transmit head coil (dStream HeadSpine coil solution, Philips AG, Zurich, Switzerland) under general anesthesia with a standardized anesthetic protocol (16).

Conventional morphological MR images included T2-weighted (T2W) turbo spin-echo sequences in transverse, dorsal, and sagittal planes, a transverse fluid-attenuated inversion recovery (FLAIR), a transverse T2^*^ or a susceptibility-weighted sequence, and a 3D T1W gradient echo sequence.

In the dogs with idiopathic epilepsy, the 3D T1W sequence was repeated after intravenous injection of contrast media [Gadodiamide (Omniscan) GE HealthcareAG, Glattbrugg, Switzerland, or Gadoteric-acid (Dotarem), Guerbet AG, Zürich, Switzerland].

The echo-planar DTI sequence was performed in a transverse plane (TR 8,191 ms; TE 81 ms) with 32 diffusion directions (single low b value = 0 s/mm^2^; maximal b value = 800 s/mm^2^; isometric voxel size of 1.5 mm, in-plan field of view of 160 × 160 mm, and acquisition matrix of 108 × 105).

### 2.3 Postprocessing

DICOM images were converted to 4D NIFTI format using dcm2niix (University of South Carolina, South Carolina, USA). Further processing was performed using FSL (FMRIB Software Library v6.0.5.1, Oxford, UK).

Using FSL commands, data were corrected for eddy current and motion distortion, and an automated mask was used to remove extraneural tissues. Then, a diffusion tensor model was fitted to the processed images using the FSL “dtifit” command, which provides a matrix-valued tensor for each voxel. Diffusion tensor maps for each diffusivity parameter were generated for each subject and visually inspected to ensure the quality of the preprocessing, volume registration, and orientation.

### 2.4 Tract-based spatial statistics

A modified tract-based spatial statistical (TBSS) analysis (21, 22) adapted to dogs (10) was conducted. As previously described by Barry et al. (10), the subjects' FA images were processed according to the human TBSS pipeline until step three (tbss_3_postreg).

Each subject's FA image was registered using a non-linear transformation to the target FA image (the most representative subject identified in step three of the human TBSS pipeline) with FNIRT (23). A mean FA image was created by concatenating the target space FA images into a single 4D file that was then averaged using *fslmaths*. A mean FA skeleton was created by thresholding the mean FA at a lower threshold of 0.2 and an upper threshold of 0.8. This thresholded FA skeleton was then binarized to create an FA skeleton mask. The skeleton mask was then applied to a 4D FA file to create a 4D FA skeleton image. MD was processed according to the same steps outlined above and extracted using the FA skeleton. FA and MD values at the location of the FA skeleton mask were then exported for statistical analysis.

### 2.5 Region of interest analysis

ROI analysis was performed for two regions compromised across all epilepsy syndromes in humans, the corpus callosum and the cingulate white matter (5). The ROIs were selected in the mean FA skeleton mask overlaid with a T1W image of the target dog allowing visualization of the corpus callosum and cingulate white matter. The created mask included the cingulate region bilaterally (Supplementary Figure 1). The corpus callosum mask was created following Barry et al. (10), but with all regions of the corpus callosum in a single mask (Supplementary Figure 2). Using *fslstats*, these ROIs were then applied to the 4D FA skeleton and to the 4D MD skeleton image (24).

### 2.6 Statistics

For a voxel-based TBSS analysis, permutation testing using FSL's *randomize* tool was used to conduct an independent *t*-test to evaluate differences in diffusion metrics between the idiopathic epilepsy group and the healthy control group using both threshold-free cluster enhancement and family-wise error correction to control for multiple comparisons (25–27).

For the ROI analysis, statistics were performed using R (2023.06.0 in RStudio) (28). In the first step, a Kruskal–Wallis test was performed to investigate differences in FA and MD values in the corpus callosum ROI and the cingulate ROI for two distinct groups: the idiopathic epilepsy group and the healthy control group. In a subsequent step, a Kruskal–Wallis test was conducted to examine the differences in FA and MD values in the corpus callosum ROI and the cingulate ROI across three distinct groups: the idiopathic epilepsy group, the healthy control group with a first-degree relative with idiopathic epilepsy, and the healthy control group with no family history of idiopathic epilepsy. *Post-hoc* pairwise comparisons among the groups were carried out using the Dunn test. To mitigate the issue of multiple comparisons, the Bonferroni correction was applied to adjust the *p*-values. In the event of statistical significance, an effect size (r) was computed to quantify the magnitude of differences observed between the group means. Overall, *p* < 0.05 was considered statistically significant.

## 3 Results

### 3.1 Study population

A total of 59 dogs were prospectively enrolled. Nine dogs were excluded because of insufficient imaging quality. A total of 50 dogs were included in the data analysis. Population characteristics are given in Table 1. In the healthy control group, all Greater Swiss Mountain dogs, and all but one Border Collie had a first-degree relative with idiopathic epilepsy. Information regarding the seizure semiology is reported in Table 2. Unfortunately, in cases with focal onset, the owners could not reliably report the side of the focal onset in most cases.

**Table 1:** Population characteristics.

	**Dogs with idiopathic epilepsy (*n =* 26)**	**Healthy controls (*n =* 24)**	**Healthy controls with a first-degree relative with idiopathic epilepsy (*n =* 12)**	**Healthy controls with no family history of idiopathic epilepsy (*n =* 12)**
**Breed**				
Beagle	0	11	0	11
Border collie	15	5	4	1
Greater Swiss Mountain dog	11	8	8	0
**Sex**				
Male	14	10	4	6
Male castrated	4			
Female	5	14	8	6
Female spayed	4			
Ratio male:female	2:1	5:7	1:2	1:1
**Bodyweight**				
Kilograms [median; range]	22.8; 14.0–70.0	19.0; 9.6–54.8	38.3; 16.4–54.8	16.4; 9.6–21.8
**Age**				
Years [median; range]	3.0; 1.0–8.6	5.6; 1.3–8.6	5.6; 2.5–8.6	5.2; 1.3–7.2

**Table 2:** Seizure semiology.

**Medical treatment at the time of MRI**		**[n]**
Phenobarbital		9
Potassium bromide		4
Levetiracetam		3
Imepitoin		1
Type of therapy	Mono	2
	Double	5
	Triple	2
**Time between first seizure and MRI**		**[n]**
< 1 month		5
>1–3 months		12
>3–12 months		6
>12 months		3
**Time between last seizure and MRI**		**[n]**
>2 days−1 week		11
>1 week−2 weeks		10
>2 weeks−4 weeks		1
>4 weeks		4
**Seizures**		**[n]**
Status epilepticus		18
Cluster seizures		9
**Seizure semiology**		
Tonic-clonic		24
Tonic		2
Focal onset secondary generalization		14
Unknown onset		8
Additional focal seizures		10
Autonomic signs	Salivation	9
	Urination	11
	Defecation	5
Postictal aggression		3
**Inter-ictal behavioral changes**		**[n]**
Anxiety		3

### 3.2 Voxel-based analysis with TBSS

**Figure 2:**
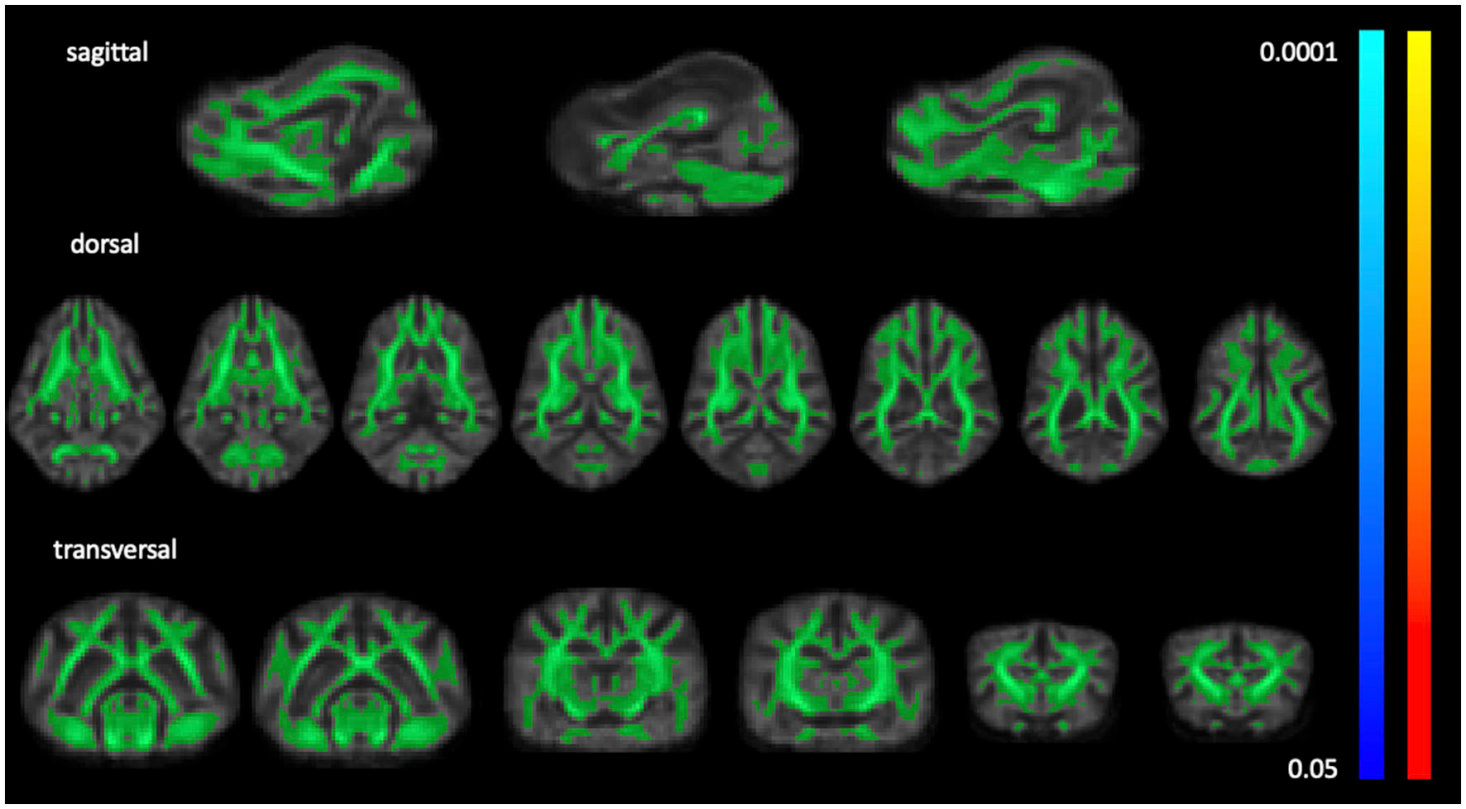
Light green overlay of the average white matter mask over sample FA template slices in sagittal, dorsal, and transverse planes. TBSS detected no significant differences from *p* < 0.05 to *p* < 0.0001 for FA (coolmap) and MD (heatmap).

No significant differences in FA and MD were identified between the idiopathic epilepsy group and the healthy control group using TBSS. The results from TBSS are displayed in Figure 2.

### 3.3 ROI analysis

**Figure 3:**
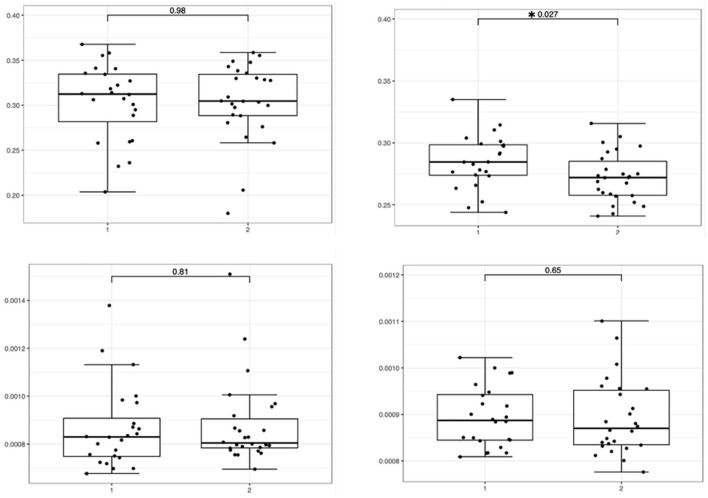
Boxplots featuring bilateral FA and MD differences in the corpus callosum and cingulate white matter with significance levels highlighted with an asterisk featuring the relevant *p*-value. The boxes on the **left side** represent the healthy controls (1), and the boxes on the **right side** (2) represent dogs with idiopathic epilepsy.

The ROI analysis showed a significant difference in the FA of the cingulate white matter in the idiopathic epilepsy group compared to the healthy control group (*p* = 0.027) with lower FA in the idiopathic epilepsy group. The effect size was moderate (r = −0.313). No significant differences were found in the FA of the corpus callosum and MD of the corpus callosum and the cingulate white matter between the idiopathic epilepsy group and the healthy control group. The results are displayed in Figure 3.

The Kruskal–Wallis test of FA and MD values of the corpus callosum ROI and the cingulate ROI across the three distinct groups revealed a significant difference in the cingulate FA and MD values (*p* = 0.012 and *p* = 0.001) but not in the callosal FA and MD values (*p* = 0.644 and *p* = 0.122).

For FA of the cingulate white matter, a pairwise comparison of the healthy control group with no familiar history of idiopathic epilepsy and the idiopathic epilepsy group showed a significant difference (*p* = 0.009) with a lower FA in the idiopathic epilepsy group (effect sizes r = −0.48).

**Figure 4:**
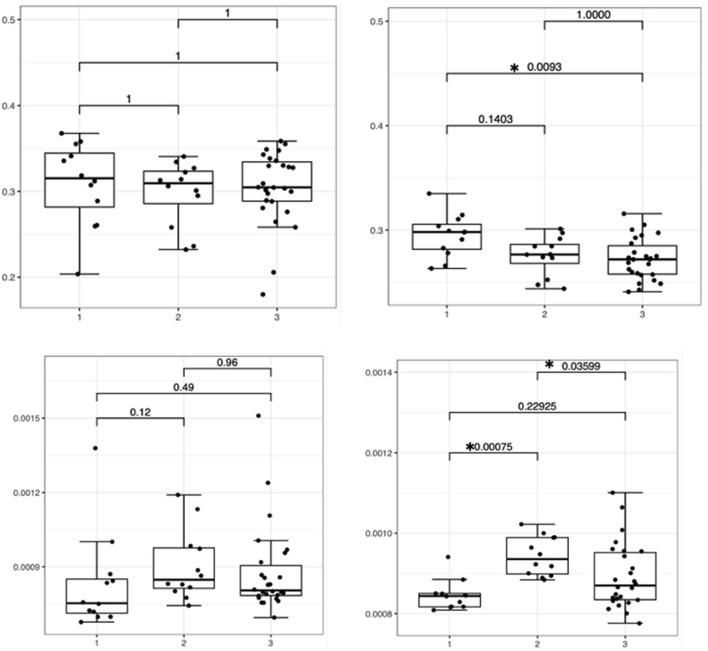
Boxplots featuring bilateral FA and MD differences in the corpus callosum and cingulate white matter with significance levels highlighted with an asterisk featuring the relevant *p*-value. Boxes on the **left side** indicate healthy controls with no familiar history of epilepsy (1), boxes in the **middle** indicate healthy controls with a first-degree relative with idiopathic epilepsy (2), and boxes on the **right side** indicate dogs with idiopathic epilepsy (3).

For MD of the cingulate white matter, a pairwise comparison of the healthy control group with no familiar history of idiopathic epilepsy and the healthy control group with a first-degree relative with idiopathic epilepsy showed significant difference (*p* = 0.0007) with a large effect size (r = −0.748) as well as a significance between the healthy control group with a first-degree relative with idiopathic epilepsy and idiopathic epilepsy group (*p* = 0.036) with a moderate effect size (r = −0.408). The results are displayed in Figure 4.

## 4 Discussion

In this single-center prospective study, we investigated white matter integrity in Border Collies and Greater Swiss Mountain dogs with idiopathic epilepsy. We hypothesized that we would find impaired white matter integrity in dogs with idiopathic epilepsy compared to healthy controls manifesting as a decrease in FA and an increase in MD. While a voxel-based analysis (TBSS) did not detect any significant differences between the idiopathic epilepsy group and the healthy control group, the ROI analysis of the corpus callosum and cingulate white matter showed significant differences in the FA in the cingulate white matter between the idiopathic epilepsy group and the healthy control group only, with lower FA values in the idiopathic epilepsy group.

Note that in general voxel-based analysis and ROI analysis in DTI do correlate well, but they do not always provide the same results (29). Automated voxel-based analysis offers the advantage that no prior assumption needs to be performed and the whole brain can be analyzed. However, in our study, the whole brain TBSS includes all 73,713 voxels in the analysis. The analysis is performed with a multiple comparison correction to prevent false-positive results. If the effect is very subtle, these multiple comparison corrections may mask small changes. In contrast, the ROI analysis first combines the signal across multiple voxels within the ROI (645 voxels for the cingulate gyrus ROI and 316 voxels for the corpus callosum ROI) leading to an increased signal-to-noise ratio. Second, as only two analyses are performed in the ROI analysis, the effect of multiple comparisons correction is minimal. Furthermore, voxel-based analysis heavily depends on the exact anatomical registration of each voxel to the study template, and the more diverse shape of the canine brain and a not as well validated processing pipeline might have had an influence on the voxel registration and therefore on the voxel-based analysis (29).

In contrast to canine epilepsy, plenty of DTI studies have been performed in human epilepsy and microstructural compromises of the white matter have been identified in a wide variety of epilepsy syndromes using DTI (5). Decreased FA and increased MD were seen across all epilepsy syndromes in a variety of white matter structures including the corpus callosum and cingulum (5).

While this effect has been very clearly identified in the large-scale ENIGMA human epilepsy study, including more than 1,000 individuals, the results from smaller studies with fewer patients have been less conclusive (5, 30, 31). In smaller studies like ours, with smaller sample sizes, it is more difficult to account for a heterogenic study population and it is more difficult to compensate for additional contributing factors, such as lateralization of the diseases, age, or gender. For example, we did not have information regarding lateralization of the seizure signs in most dogs included in the study, and gender and age were not matched between the idiopathic epilepsy group and the healthy control groups. Furthermore, a stringent classification of epilepsy into a subcategory was lacking in our canine patients. All these factors might have led to the detection of reduced FA in the cingulate white matter only.

In humans, the DTI changes were most pronounced in ipsilateral to mesial temporal lesions and the changes were less pronounced in non-lesional epilepsy (5). We included dogs with generalized epileptic seizures and no visible lesions on MRI, where the effect might be less pronounced compared to focal lesional epilepsy syndromes in humans.

Furthermore, most dogs included in our study were newly diagnosed. In humans, it has been speculated that the white matter changes represent secondary effects, rather than being causal, but large longitudinal studies are lacking (5). Therefore, enrolling dogs with idiopathic epilepsy at the first stages of clinical manifestation of the disease might have had an impact on the ability to detect white matter microstructural abnormalities, and eventually, long-term follow-up examinations could lead to different results.

Previously, inter-ictal brain diffusion in canine idiopathic epilepsy has been investigated with simple diffusion-weighted imaging and a ROI-based approach only. Gray and white matter ROIs have been investigated, but neither the corpus callosum nor the cingulate white matter were included (9). Increased ADC values in dogs with idiopathic epilepsy have been identified in the semioval center and the piriform lobe in this study (9). In contrast to this former study, with our ROI-based approach, we have focused on the white matter structures most commonly involved in human epilepsy (corpus callosum and cingulate white matter). Additionally, we have performed a voxel-based whole-brain approach, which did not depict increased MD in the semioval center or in the piriform lobe.

A more recent retrospective multicentric study focused on peri-ictal imaging findings and found mixed ADC results, including normal, increased, and decreased diffusivity in affected areas (32). The cingulate gyrus was affected in 6/19 cases and had either decreased ADC values in the cortex and increased ADC values in the white matter, or increased ADC values in the white matter only (32). The cingulate gyrus was commonly affected by peri-ictal imaging changes, which supports the cingulate gyrus as a target area in dogs with idiopathic epilepsy. However, in our study, we investigated MD as a measure for overall diffusion and could not detect any significant difference in the MD, but significantly reduced FA in dogs with idiopathic epilepsy compared to healthy controls in the cingulate white matter. While both studies suggest the cingulate white matter as a target structure in canine idiopathic epilepsy, the microstructural changes might differ inter-ictally and peri-ictally and longitudinal studies including peri-ictal and inter-ictal time points could give insights into the time course of such changes.

The pathophysiology of white matter changes and its correlation with DTI changes in epilepsy is not well understood. It is still unknown whether the identified white matter abnormalities predate the development of epilepsy or whether they are a secondary effect of ongoing seizures (5). No histological examination was performed to look for a correlation between the reduced FA and possible histological changes in the brains of the dogs with idiopathic epilepsy included in our study. However, various mechanisms have been suggested, including changes related to the underlying epileptogenic process, axonal degeneration, and compensatory white matter reorganization (31, 33). Correlation between histopathological changes and DTI changes in epilepsy is sparse and is derived from rat models and surgically removed brain tissue in humans (34, 35). In the rat model of status epilepticus, myelin staining was reduced in the fimbria of the fornix in correlation with reduced FA 8 weeks after induced status epilepticus (34). In humans with temporal lobe epilepsy, increased extra-axonal fraction, and reduced cumulative axonal membrane circumference and myelin area were found in the surgically extracted fimbria of the fornix (35). Studies investigating histopathological changes in canine epilepsy are sparse (36). In contrast to humans, there is less evidence of temporal lobe involvement in dogs (36). Similar to humans with epilepsy originating from the limbic system, neuronal loss and gliosis were found in the limbic system, including the cingulate gyrus, amygdaloid nucleus, dorsal and ventral parts of the hippocampus, and dorsomedial nucleus of the thalamus in an epileptic Shetland Sheepdog family (37, 38). Involvement of the cingulate cortex would make an effect on the cingulate white matter a reasonable possibility. In contrast, Buckmaster et al. failed to identify any neuropathological changes in the temporal lobe of dogs with medically intractable epilepsy (39). In addition, guidelines for pathological examination of epileptic canine brains have been redefined (40); large studies reporting pathological abnormalities are still lacking.

We investigated idiopathic epilepsy in Border Collies and Greater Swiss Mountain dogs, two dog breeds with a suspected genetic background for epilepsy (41–43). Over the last decade, idiopathic epilepsy with a proven or suspected genetic background has been reported for a number of dog breeds with most studies focusing on clinical characteristics and genetic aspects. Nevertheless, most studies have not yet identified causative gene mutations, suggesting that inheritance may be complex (44). We have chosen Border Collies and Greater Swiss Mountain dogs because of their severe form of epilepsy and because these are commonly affected breeds in the author's institution. Choosing dog breeds with well-known epilepsy offered the advantage of more narrowly defined, more similar seizure phenotypes. Furthermore, the true prevalence of epilepsy in these breeds is currently unknown, but based on the literature, it is assumed that it is rather high (42–44). In pursuit of generating comparable data from groups of animals as homogeneous as possible, healthy first-degree relatives from the affected dogs with idiopathic epilepsy were recruited. What seemed to be an advantage when we designed the study might have had a negative impact on our results. Of the healthy Border Collies and Greater Swiss Mountain dogs included in our study, all but one had a first-degree relative with idiopathic epilepsy. In humans, heritability of the white matter microstructure has been demonstrated (45, 46), and in patients with MRI-negative temporal lobe epilepsy, an increase in MD has been identified even in asymptomatic siblings (47). Increased MD was also found in the healthy control dogs with a first-degree relative with idiopathic epilepsy compared to healthy controls without a first-degree relative with idiopathic epilepsy (Figure 4). It is therefore possible that in close relatives of dogs with suspected genetic idiopathic epilepsy, white matter could be compromised even in unaffected animals. Unfortunately, all but one healthy control dog without a first-degree relative with idiopathic epilepsy were from a different breed (Beagle dogs), and to test the concept of hereditary white matter changes, we would have needed more dogs of the same breed without a family history of epilepsy. Furthermore, the high number of close relatives included in our study may have hidden differences in MD between dogs with idiopathic epilepsy and healthy controls because of heritable white matter changes. Such heritable white matter changes could even turn out to be diffusion-based endophenotypes for epilepsy in dogs. Such diffusion-based endophenotypes might support an imaging-based diagnosis for genetic epilepsy in the future (47).

### 4.1 Limitations

One of the strongest limitations of our study is the heterogenicity of the study population. We were not able to recruit a control group with the same age and gender profile as the idiopathic epilepsy group. The median age in the control group was almost twice as high as in the idiopathic epilepsy group. Barry et al. have shown significant differences in FA and MD between dogs of <7 years and dogs of >10 years of age (10). We did not include dogs older than 10 years of age, but we cannot rule out that the different ages in both groups had an influence on the results. The gender ratio was reversed between the two groups. The idiopathic epilepsy group included mainly male dogs, while the control group included more females than males. The influence of sex on the microstructure has been proven in humans; for example, one study has shown increased FA in male participants compared to female participants in all subregions of the corpus callosum (48). Such gender-related differences could mask differences due to illness as there was a higher percentage of male dogs in the idiopathic epilepsy group compared to the control group.

We also have different breed distributions in both groups, but to date, it is not known whether there is any regional difference between FA and MD in different dog breeds.

In humans, countless DTI studies have been conducted over the last decade, and post-processing has evolved massively. Well-tested and adapted pipelines exist. In contrast, in veterinary medicine, an adapted version of the TBSS pipeline has just recently been published (10), and although we followed this pipeline in the main, we had to do without the distortion correction due to our imaging acquisition. This might be of even more compromise in dogs than in humans. Dogs have larger frontal sinuses compared to humans, which can cause problems in susceptibility-sensitive sequences (49). In DTI post-processing, it is possible to account for these susceptibility-induced field distortions (50, 51). Unfortunately, the data in our study were acquired with one B0 image only, and at least two B0 images with opposite phase encoding directions are needed for field estimation (50). Therefore, no correction for susceptibility-induced distortions was performed, and diffusion images were displayed with geometric mismatches compared to the structural images. We cannot rule out that the different degrees of distortion in the individual images potentially influenced our results.

## 5 Conclusion

We aimed to investigate white matter diffusion changes in dogs affected by idiopathic epilepsy with generalized tonic-clonic seizures. We observed subtle changes in DTI between dogs with idiopathic epilepsy and healthy controls limited to cingulate white matter, with a significantly lower FA in dogs with idiopathic epilepsy compared to healthy controls using a ROI approach. No significant changes were found between dogs with idiopathic epilepsy and healthy controls in the TBSS analysis and in the corpus callosum in the ROI approach between both groups. This study supports the cingulate area as a target structure in canine epilepsy. The subtle changes only might be explained by the small sample size and the higher variability in canine brain anatomy. Furthermore, all included dogs showed generalized tonic-clonic seizures, possibly suffering from generalized epilepsy syndrome, which is also associated with less pronounced DTI changes in humans than focal epilepsy syndromes.

## Data availability statement

The raw data supporting the conclusions of this article will be made available by the authors, without undue reservation.

## Ethics statement

The animal studies were approved by Swiss Federal Veterinary Office Zurich. The studies were conducted in accordance with the local legislation and institutional requirements. Written informed consent was obtained from the owners for the participation of their animals in this study.

## Author contributions

KB: Conceptualization, Data curation, Formal analysis, Funding acquisition, Investigation, Methodology, Project administration, Resources, Validation, Visualization, Writing—original draft, Writing—review & editing. AW-L: Methodology, Writing—review & editing, Writing—original draft. FS: Writing—review & editing. HR: Formal analysis, Project administration, Writing—review & editing. MD: Writing—review & editing. RB: Writing—review & editing. IC: Conceptualization, Methodology, Writing—review & editing. SH: Conceptualization, Investigation, Methodology, Supervision, Writing—review & editing.

## Supplementary material

Supplementary Figure 1 Sagittal, dorsal, and transverse views of the delineation of the cingulate ROI (blue) overlayed on the average white matter mask (green) over sample FA template.

Supplementary Figure 2 Sagittal, dorsal, and transverse views of the delineation of the corpus callosum ROI (purple) overlayed on the average white matter mask (green) over sample FA template.
