# Supplementary figures and images for: Diffusion tensor-based analysis of white matter in dogs with idiopathic epilepsy
Source: Front Vet Sci. 2023 Dec 18;10:1325521. doi: 10.3389/fvets.2023.1325521 (PMC10773822; doi:10.3389/fvets.2023.1325521)

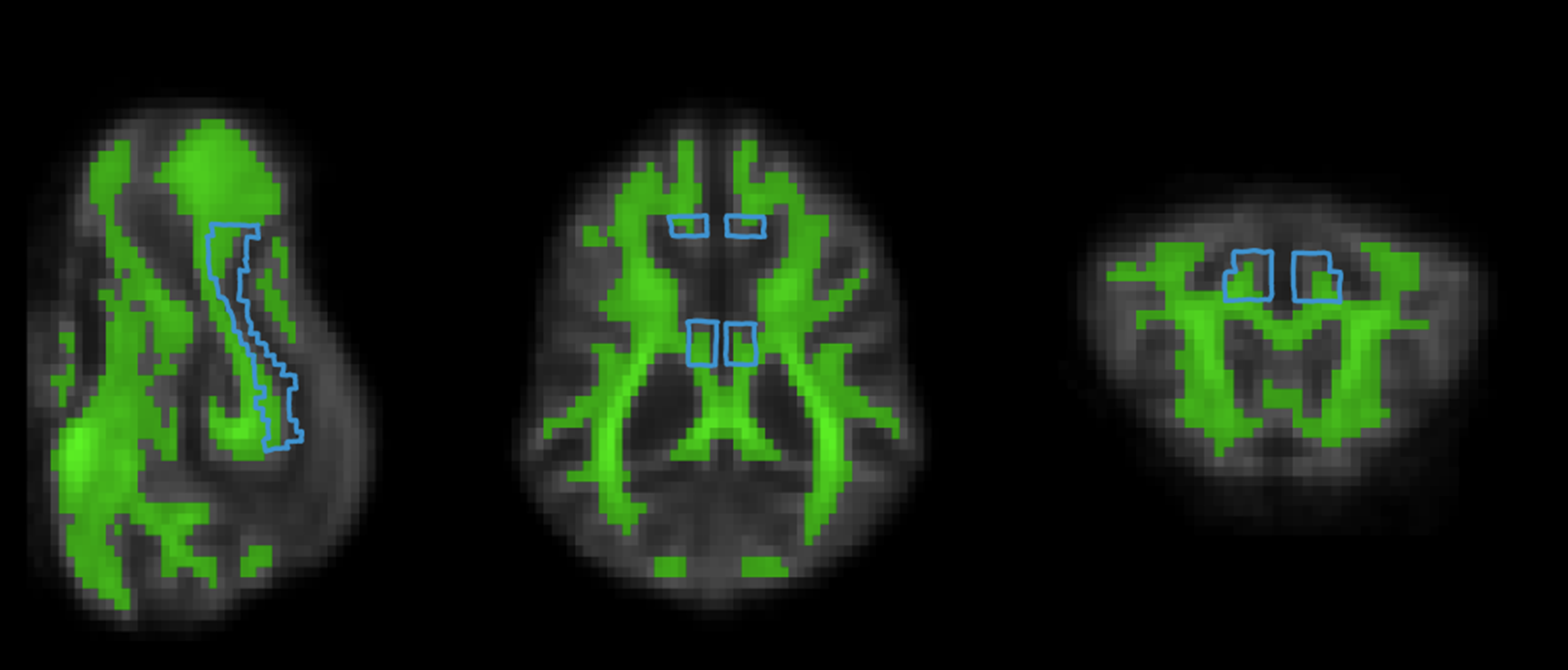

Supplement: Supplementary Figure 1 — Sagittal, dorsal, and transverse views of the delineation of the cingulate ROI (blue) overlayed on the average white matter mask (green) over sample FA template. [file Image_1.TIFF]

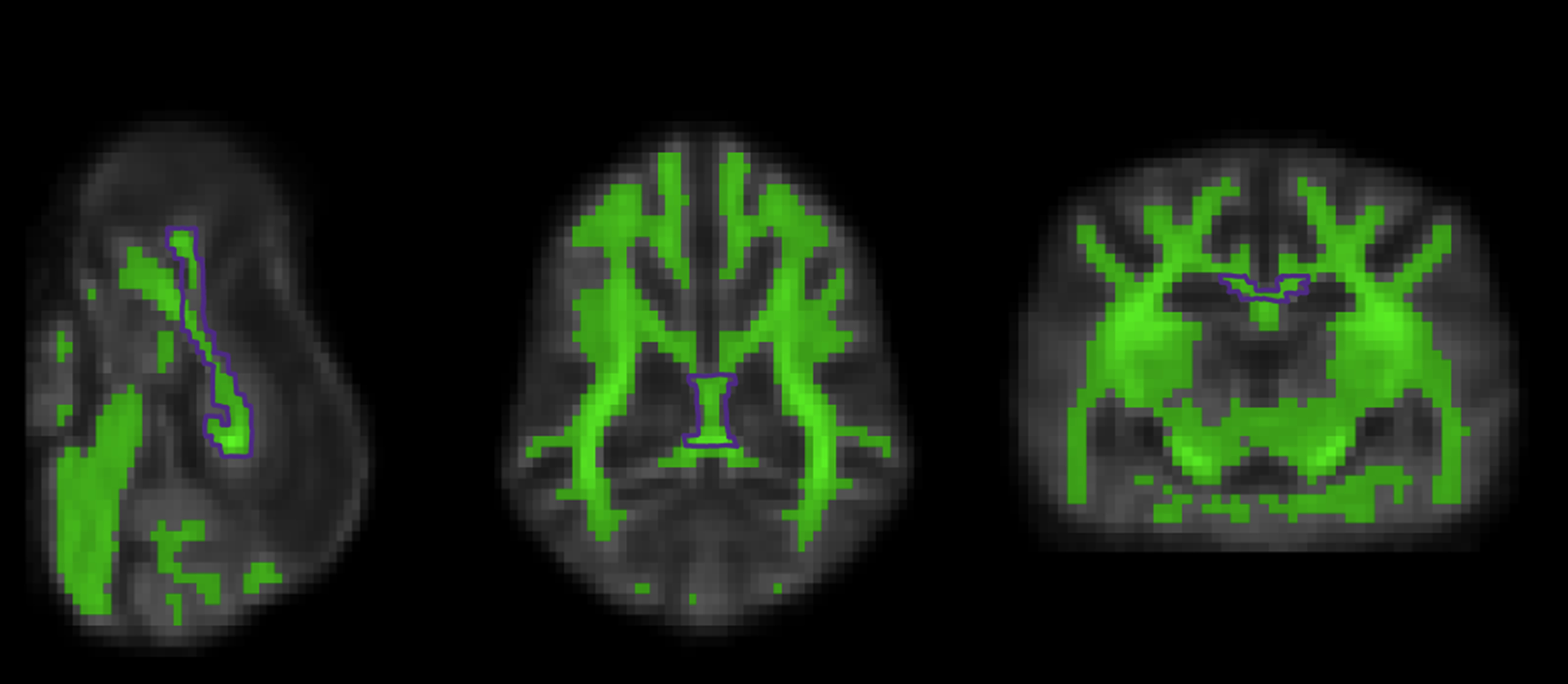

Supplement: Supplementary Figure 2 — Sagittal, dorsal, and transverse views of the delineation of the corpus callosum ROI (purple) overlayed on the average white matter mask (green) over sample FA template. [file Image_2.TIFF]
